# Beneficial Anti-Platelet and Anti-Inflammatory Properties of Irish Apple Juice and Cider Bioactives

**DOI:** 10.3390/foods10020412

**Published:** 2021-02-12

**Authors:** Alexandros Tsoupras, Donal Moran, Hayley Pleskach, Maria Durkin, Con Traas, Ioannis Zabetakis

**Affiliations:** 1 Department of Biological Sciences, University of Limerick, V94 T9PX Limerick, Ireland; Donal.Moran@ul.ie (D.M.); hayleypleskach@hotmail.com (H.P.); 16159195@studentmail.ul.ie (M.D.); Con.Traas@ul.ie (C.T.); Ioannis.Zabetakis@ul.ie (I.Z.); 2 Health Research Institute, University of Limerick, V94 T9PX Limerick, Ireland; 3 Bernal Institute, University of Limerick, V94 T9PX Limerick, Ireland

**Keywords:** CVD, platelets, polar lipids, fermentation, apple, cider, PUFA, MUFA, PAF, ADP

## Abstract

Several bioactives from fruit juices and beverages like phenolics, nucleotides and polar lipids (PL) have exhibited anti-platelet cardio-protective properties. However, apple juice and cider lipid bioactives have not been evaluated so far. The aim of this study was to investigate the anti-platelet and anti-inflammatory effects and structure activity relationships of Irish apple juice and Real Irish cider lipid bioactives against the platelet-activating factor (PAF)- and adenosine diphosphate (ADP)-related thrombotic and inflammatory manifestations in human platelets. Total Lipids (TL) were extracted from low, moderate and high in tannins apple juices and from their derived-through-fermentation cider products, as well as from commercial apple juice and cider. These were separated into neutral lipids (NL) and PL, while all lipid extracts were further assessed for their ability to inhibit aggregation of human platelets induced by PAF and ADP. In all cases, PL exhibited the strongest anti-platelet bioactivities and were further separated by high-performance liquid chromatography (HPLC) analysis into PL subclasses/fractions that were also assessed for their antiplatelet potency. The PL from low in tannins apple juice exhibited the strongest antiplatelet effects against PAF and ADP, while PL from its fermented cider product were less active. Moreover, the phosphatidylcholines (PC) in apple juices and the phosphatidylethanolamines (PE) in apple ciders were the most bioactive HPLC-derived PL subclasses against PAF-induced platelet aggregation. Structural elucidation of the fatty acid composition by gas chromatography mass spectra (GCMS) analysis showed that PL from all samples are rich in beneficial monounsaturated fatty acids (MUFA) and omega 3 (n-3) polyunsaturated fatty acids (PUFA), providing a possible explanation for their strong anti-platelet properties, while the favorable low levels of their omega-6/omega-3 (n-6/n-3) PUFA ratio, especially for the bioactive PC and PE subclasses, further support an anti-inflammatory cardio-protective potency for these apple products. In conclusion, Irish apple juice and Real Irish cider were found to possess bioactive PL compounds with strong antiplatelet and anti-inflammatory properties, while fermentation seems to be an important modulating factor on their lipid content, structures and bioactivities. However, further studies are needed to evaluate these effects.

## 1. Introduction

Specific foods and beverages included in a healthy diet contain natural bioactive compounds with health benefits and especially for preventing several inflammation-related chronic pathologies [1,2]. For example, phenolic compounds and bioactive polar lipids (PLs) found in foods and beverages like olive oil, fish and red and white wine have demonstrated cardio protective properties by reducing or preventing inflammation [1,2,3].

Apples and apple products like apple juice and cider have been proposed to belong in this category of healthy foods/fruits and beverages, respectively. Apples are a versatile, widely available and highly nutritional fruit, so unsurprisingly are one of the top consumed fruits in the world. Cider is an ancient beverage made from the fermented juices of apples. It has an alcohol content ranging between 1.2% and 8.5% ABV (alcohol by volume) [4], lower than wine due to the lower sugar content of apples compared to grapes. Fermentation is the basis for cider production too, while the composition of apple cider is affected by the variety of apple, growth conditions, apple cultivar, ripening stage, fermentation type, yeast strains, equipment and method, and the use of additives [5].

Apple cider has been characterized as a functional beverage due to its being rich in bioactive polyphenols content like apples, but also due to its natural microbiom-probiotics that beneficially affects the gut microbiota. Thus, the health benefits associated with apples, and their fermented products like cider, are possibly attributed to their high-in-bioactives content, since they are abundant in minerals, potassium and magnesium, and a rich source of predominantly vitamin C and E, high in phytochemicals including triterpenoids, such as ursolic acid, and polyphenols such as flavonoids and beneficial microbiom-probiotics [6,7].

However, there is no evidence to date for the potential health benefits of the lipid content of apples and its products like apple juices and apple cider. In other beverages that are also derived from fermentation of plant/fruit sources, such as red and white wine and relative musts and yeasts for their production, as well as beer, they have all been found to contain bioactive PL like phospholipids and glycolipids with cardio-protective abilities against the inflammatory and thrombotic mediator platelet-activating factor (PAF) and its associated pathways [8]. The same was also shown in beers that were found to contain specific PL subclasses and molecular species with beneficial bioactivities against inflammation and thrombosis, as a result of fermentation, in comparison to their raw materials [9].

PAF is a key lipid mediator involved in inflammation, thrombosis and thrombo-inflammatory manifestations of several inflammation-related chronic diseases [2,3], while bioactive PL compounds found in healthy foods and in beverages like red/white wine and beer can reduce the intensity of thrombosis and inflammation and prevent chronic disorders by reducing the levels and activities of PAF and its associated pathways of inflammatory and thrombotic/thrombo-inflammatory manifestations [1,2,3]. In addition, structure activity relationship studies have also revealed that bioactive PLs in such foods/beverages were found to have structural analogy with the structures of the classic PAF molecule, and hence had capabilities to carry out strong antagonistic effects on its receptors in several cells involved in inflammation and thrombosis, including platelets [2,9]. Subsequently, the presence of such bioactive PLs in these foods and beverages has provided beneficial preventative properties against inflammation and platelet aggregation—thrombosis-related disorders [1,2,3].

Moreover, the water-soluble part of apples was previously found to have little to no success against platelet aggregation and associated manifestations induced by classic platelet agonists like adenosine diphosphate (ADP) and collagen [10], while, as aforementioned, there has been no evidence to date of the antiplatelet potential of the lipid content in apple products like apple juice and cider. Therefore, the purpose of this study was to investigate the antiplatelet potential of the lipid content of Irish apple products, such as Irish apple juice and its fermented Real Irish cider against platelet aggregation induced by the thrombotic and inflammatory mediators, like PAF, and by classic platelet agonists, like ADP, and to evaluate the fatty acid content and structures and structure activity relationships via gas chromatography mass spectra (GCMS) of the most bioactive lipids separated by high-performance liquid chromatography (HPLC) analysis. The apple juice from different varieties of apples, including Jonagold, Dabinett, and Aston Bitter, which vary in tannin content, were used in this investigation as the lipid content has been shown to vary across different varieties [11]. Both the apple juices from these apples and cider derived from them were examined and compared with retail apple juice, whose composition included the Jonagold variety, and Irish Cider, whose composition included all three apple varieties.

It should also be stressed that ciders can legally be made with 75% or more added water (depending on the regulations in force in particular countries), as well as the addition of extra sugar, thus, in the final product, the bioactive components are usually diluted, whereas the fresh and the retail Real Irish Ciders that were chosen to be studied are ciders made using only apple juice, without the addition of water or sugar.

## 2. Materials and Methods

### 2.1. Materials, Reagents and Instrumentation

Materials for platelet aggregation were purchased from Labmedics LLP (Abingdon on Thames, UK). ADP was purchased from Chronolog (Havertown, PA, USA). Standard PAF and bovine serum albumin (BSA) were purchased from Sigma Aldrich (Wicklow, Ireland). For the sampling of blood, 20G safety needles and evacuated sodium citrate S-monovettes were purchased from Sarstedt Ltd. (Wexford, Ireland). Using a Chronolog-490 two channel turbidimetric platelet aggregometer (Havertown, PA, USA) coupled to the accompanying AGGRO/LINK software package, analyses of human platelet-rich plasma (hPRP) for platelet aggregation bioassays were carried out. Using an Eppendorf 5702R centrifuge (Eppendorf Ltd., Stevenage, UK), centrifugations were carried out. A Shimadzu UV-1800 spectrophotometer (Kyoto, Japan) was used with a quartz 1 cm cuvette for the spectrophotometric analysis.

An Alliance e2695 Separations Module, in tandem with a Waters 2487 UV detector and an Empower Chromatography Data Software was utilized for the separation of apple juice and cider bioactive PLs into subclasses via HPLC analysis. Using a Varian 410-Gas Chromatographer coupled to a Varian 210-MS detector equipped with a split/splitless injector (Agilent Technologies, Palo Alto, CA, USA), the GC-MS analysis was performed. The standards and reagents used for GC-MS and HPLC were supplied by Sigma Aldrich (Wicklow, Ireland). All additional glass and plastic consumables, solvents, and reagents were of analytical grade and were purchased from Fisher Scientific Ltd. (Dublin, Ireland). Flash rotary evaporation (Buchi Rotavapor, Mason Technology Ltd., Dublin, Ireland) was used for the evaporation of solvents from all lipid extracts, while Nitrogen stream from Nitrogen cylinders (BOC, Ireland) was used for evaporations in Nitrogen environment.

### 2.2. Apple Juice and Cider Samples Assessed

Apple juice and cider samples that were analysed were provided by the Irish private company ‘The Apple Farm’ Co. Tipperary. The varieties of apple juice supplied were AA for Jonagold (a low in tannins variety), AB for Dabinett (an intermediate in tannins variety) and AE for Aston bitter (a high in tannins variety). *Champagne* yeast was added, and each apple juice type was fermented through a one-month fermentation process to produce three different cider samples, CA, CB and CE, respectively. The ciders were checked regularly (by organoleptic/taste tests and pH measurements) for presence of acetic acid, which would indicate the commencement of conversion to cider vinegar by acetobacter. Standards of commercially available apple juice (relevant to the apple juice of the Jonagold variety, but pasteurized) and cider ‘Con’s Irish Cider’ from the same company (produced by fermentation of a mixture of the aforementioned three different apple juice types in a ratio of 55% Jonagold, 35% Dabinett and 10% Aston bitter) were also analysed. Triplicates (N = 3) for each sample were used for further analysis.

### 2.3. Extraction

The total lipids (TL) from each sample were extracted as previously described [9,12] based on the Bligh and Dyer extraction method [13]. Briefly, the TL extraction was achieved by homogenisation of the sample in a monophasic system containing chloroform/methanol/water in a 1:2:0.8 (*v/v/v*) ratio. Addition of appropriate volumes of water and chloroform was then performed in order to adjust the chloroform/methanol/water-based homogenate at a ratio of 1/1/0.9 (*v/v/v*) to achieve phase separation with the TL being present in the lower phase. This phase was gathered in round-bottom flasks and evaporated until dry on a flash rotary evaporator at 37 °C under vacuum between 700 and 50 mbar (Buchi Rotavapor, Mason Technology Ltd., Dublin, Ireland), and then re-dissolved in a chloroform/methanol solution at a ratio of 1/1 (*v/v*) and transferred to a small glass tube, which was evaporated under nitrogen stream. The obtained TL were then weighted and stored under nitrogen at −20 °C for a maximum of 8 weeks.

The obtained TL extracts of all the samples were then further separated into their PL and Neutral Lipids (NL) fractions as previously described [9,12], based on the counter-current distribution method of Galanos and Kapoulas [14]. Pre-equilibrated petroleum ether and 87% ethanol were used to obtain the NL and the PL extracts, with completion of this method yielding the PL in the ethanol phase and the NL in the petroleum ether phase within a separatory funnel Briefly, the TL were dissolved in 2 mL of an hydroalcoholic solvent of 87% ethanol and this solution containing the TL was transferred in a separatory funnel containing 30 mL of petroleum ether. After a phase separation occurred, the lower ethanolic phase was extracted into a second separatory funnel containing another 30 mL of pre-equilibrated petroleum ether to ensure further separation of any remnants of NL from the ethanolic phase that contained the PL. These steps were repeated several times until all samples had been washed through, leaving the PL in the 87% ethanol phases and the NL in the petroleum ether phases, respectively. Both phases were collected in round-bottom flasks and evaporated using a rotary evaporator until dry.

When the TL, NL, and PL extracts were obtained, they were then evaporated, weighed and stored for further analysis under a nitrogen atmosphere at −20 °C.

### 2.4. Platelet Aggregometry Biological Assays

The evaluation of the antithrombotic properties of all TL, PL, and NL extracts from all apple juice types, ciders and their fractions derived from their HPLC analysis were performed in human platelet-rich plasma (hPRP) preparations from healthy donors as previously described [12,15], to assess their ability against aggregation of human platelets induced by the inflammatory and thrombotic mediator PAF and by the well-established platelet agonist ADP. The 50% inhibitory concentration value—IC50 value (half-maximal inhibitory concentration) for each sample was calculated by the amount (μg) of the lipid sample that led to 50% of inhibition of human platelet aggregation induced by PAF or ADP in hPRP platelet suspensions of 0.250 mL. The resulting IC50 values were expressed as a mean value of the mass of lipid (μg) in the aggregometer cuvette ± standard deviation (SD). Using blood samples from different donors, all experiments for evaluating the bioactivities of each lipid extract from each apple juice/cider sample were performed several times (n = 6), for each replicate, in order to ensure reproducibility.

### 2.5. High-Performance Liquid Chromatography

**Figure 1:**
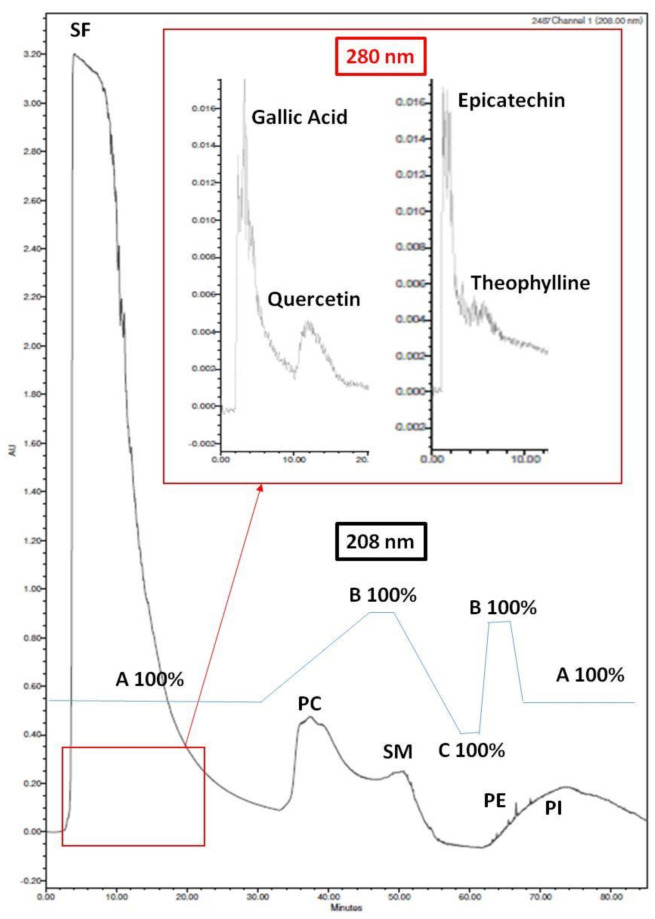
Representative Chromatogram of standards analysed by the HPLC method in 208 nm (Polar Lipids) and 280 nm (Phenolic compounds). Abbreviations: PC: Phosphatidylcholine; SM: Sphyngomyelin; PE: Phosphatidylethanolamine; PI: Phosphatidylinositol; SF: Solvent Front; A: Acetonitrile; B: Methanol; C: Water.

The most bioactive PL extracts were further separated by HPLC analysis into PL subclasses. A normal phase column, Luna 10 μm NH2 100A, LC Column 250 × 4.6 mm, Ea from Phenomenex (Hurdsfield, UK), was used. The solvent system contained an isocratic elution with 100% solvent A (acetonitrile) for 30 min followed by a linear gradient to 100% solvent B (methanol) in 15 min, a hold for 5 min in 100% solvent B, followed by a linear gradient to 100% solvent C (water) in 10 min and a hold in the same solvent for 3 min, followed again by a linear gradient to 100% solvent B for 3 min and a hold for 4 min in 100% of the same solvent, followed by a linear gradient to 100% solvent A for 5 min, and finally a hold in 100% solvent A for 10 min. Standard solutions at volumes of 5–20 μL, which contained 20–100 mg of PL (Sigma Aldrich), were injected each time. The flow rate was 1 mL/min and the eluted substances detected spectrophotometrically, using UV detection at 208 nm for phospholipids and glycolipids and 280 nm for phenolics as shown in Figure 1 and Table 1.

According to the retention times obtained for these standards, during similar HPLC analysis of the PL extracts from all apple juices and cider samples, six fractions were collected as follows: Fraction 1, 0–15 min; Fraction 2, 15–30 min; Fraction 3, 30–45 min; Fraction 4, 45–60 min; Fraction 5, 60–75 min; Fraction 6, 75–90 min.

### 2.6. Gas Chromatography–Mass Spectrometry (GC–MS)

The fatty acid methyl esters (FAME) for the ciders’ and apple juices’ lipid samples (PL extracts and bioactive lipid fractions derived from the HPLC analysis of specific PL extract) were prepared and analysed by GC–MS according to the method of [12].

### 2.7. Statistical Analysis

Using Kolmogorov–Smirnov criterion, normality for all IC50 values and FA composition acquired for each lipid sample was tested. After this, all comparisons of IC50 values against ADP- and PAF-induced platelet aggregation were observed using one-way analysis of variance (ANOVA). Comparisons in the lipid content and fatty acid (FA) composition acquired from the GC–MS analysis were observed using the Kruskal–Wallis nonparametric multiple comparison test. For p-values less than 0.05 (*p* < 0.05) differences were considered statistically significant. The data were analyzed using a statistical software package (IBM-SPSS statistics 25 for Windows, SPSS Inc., Chicago, IL, USA).

## 3. Results and Discussion

### 3.1. Yield of Extraction

The yield of extraction for the recovered amounts of TL and its separated sub-fractions of PL and NL (expressed as g of lipids/100 g of sample) from all samples, the 3 raw apple juices AA, AB and AE, 3 raw apple ciders CA, CB and CE, as well as the commercial controls of apple juice AD and cider CD, respectively, are shown in Table 2.

The yield of TL extract in these apple-based beverage samples (apple juice and cider) was found to be of similar yield to other plant-derived beverages such as wine, beer and tea [8,9,16,17], but lower from other food sources that are rich in lipids, such as dairy and marine-related sources [12,18,19].

Of the PL and NL fractions retrieved from the TL extracts, there were significantly higher amounts present of PL, ranging from 80 to 95% of the TL, in comparison to NL ranging from 5 to 20% of the TL in all samples except the commercial apple juice sample where the PL and NL were of approximately the same magnitude. Thus, the yield for PL approximately ranged from 0.015–0.090 g/100 g of the apple beverages, apart from the pasteurized commercial apple juice that exhibited much higher yield (approximately 0.2 g/100 g). These results come also in accordance with previously reported ones for the yield of PL from other plant-derived beverages such as wine, beer and tea [8,9,16,17]. A higher yield was noted in apple juices AD and AE, while apple juice AB had the lowest amount of TL and therefore PL extracts. Similar findings were observed in cider products, with cider B showing the lowest yield. However, the yield of extract for the apple juice samples was seen to be higher than the cider extracts, excluding the commercial cider sample where it had a similar yield of TL and PL to that of apple juice B, the lowest yield observed in the apple juice samples. It therefore can be considered that some lipids are utilized throughout fermentation during the development of cider. Moreover, pasteurized commercial apple juice exhibited much higher yield than all the other apple-based beverages, suggesting that more lipids were released due to the increased temperature used for pasteurization and thus more lipids were found in its extracts.

### 3.2. Anti-Platelet Effects of Apple Juice and Cider Lipids

**Figure 2:**
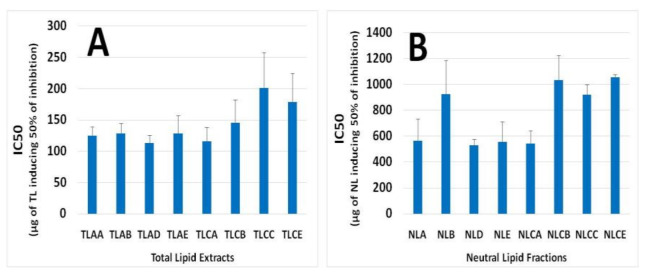
Anti-platelet activities (IC50 values) of TL (**A**) and NL (**B**) extracts from different apple juice (AA, AB, AD and AE) and cider samples (CA, CB, CC and CE) against PAF-induced human platelet aggregation. Abbreviations: TL: Total lipids; NL: Neutral lipids; AA and CA: apple juice and cider from apples with low content in tannins (Jonagold), respectively; AB and CB: apple juice and cider from apples with moderate content in tannins (Dabinett); AE and CE: apple juice and cider from apples with high content in tannins (Aston Bitter); AD: commercially found apple juice (relevant to the apple juice AA of the Jonagold variety); CC: commercially found cider ‘*Con’s Irish Cider*^®^’ (produced by fermentation of a mixture of the aforementioned three different apple juice types in a ratio of 55% Jonagold, 35% Dabinett and 10% Aston bitter).

**Figure 3:**
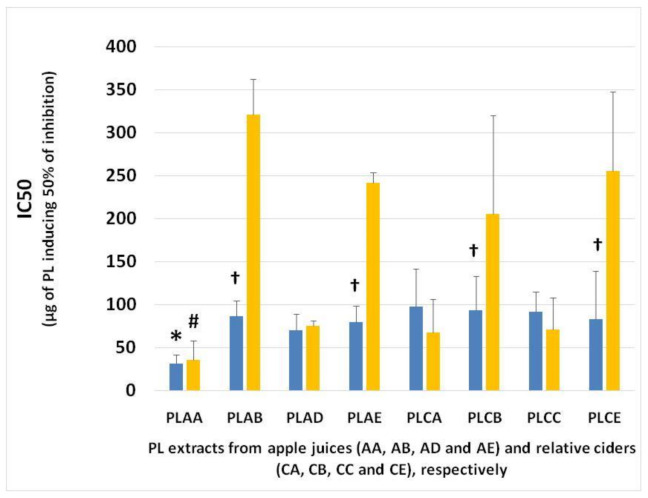
Antiplatelet activities of PL extracts from different apple juice and cider samples against human platelet aggregation induced by PAF and ADP. The blue bars represent the IC50 values of PL extracts against the PAF pathway, and the yellow bars indicate the IC50 values against ADP pathway of platelet aggregation. * denotes statistically significant difference (*p* < 0.05) of the anti-PAF potency (IC50 value) of apple juice AA in comparison to the relative IC50 values of PL from all the other samples tested against PAF-induced aggregation of platelets. # denotes statistically significant difference (*p* < 0.05) of the anti-ADP potency (IC50 value) of apple juice AA in comparison to the relative IC50 values of PL from all the other samples tested against ADP-induced aggregation of platelets. † denotes statistically significant difference (*p* < 0.05) of the anti-PAF potency (IC50 value against PAF) of a sample in comparison to its anti-ADP effects (IC50 value against ADP). Abbreviations: PL: polar lipids; AA and CA: apple juice and cider from apples with low content in tannins (Jonagold), respectively; AB and CB: apple juice and cider from apples with moderate content in tannins (Dabinett); AE and CE: apple juice and cider from apples with high content in tannins (Aston Bitter); AD: commercially found apple juice (relevant to the apple juice AA of the Jonagold variety); CC: commercially found cider ‘*Con’s Irish Cider*^®^’ (produced by fermentation of a mixture of the aforementioned three different apple juice types in a ratio of 55% Jonagold, 35% Dabinett and 10% Aston bitter).

The in vitro anti-platelet activities of all lipid samples (TL, NL and PL), extracted from apple juices AA, AB, AD and AE and from the fermented cider products CA, CB, CC and CE, were evaluated by their inhibitory effects against human platelet aggregation induced by the inflammatory and thrombotic mediator PAF or by the well-established platelet agonist ADP. The results are presented in Figure 2 for TL and NL and Figure 3 for PL, expressed as their IC50 value against PAF/ADP. This is a measure of the mass of lipid, in micrograms (μg), required to induce half maximal-reversible aggregation of human platelets in the presence of a thrombotic mediator such as PAF or a platelet agonist such as ADP. A lower IC50 value indicates a high ability of inhibition against PAF- or ADP- induced platelet aggregation.

The PL from all samples have shown to be the most bioactive lipid samples, with IC50 values being approximately within the range of 20–120 μg against the PAF pathway (Figure 3). The TL samples showed an intermediate but considerable antithrombotic potency against PAF-induced human platelet aggregation, with IC50 values being approximately within the range of 100–200 μg, as displayed in Figure 2A, while NL samples showed poor inhibitory action, with IC50 values being approximately within the range of 500–1000 μg, as displayed in Figure 2B.

These results come also in accordance with previously reported ones for similar differences observed in the anti-platelet properties against PAF of TL, NL and PL extracts from other plant-derived beverages such as wine, beer and tea [8,9,16,17]. More specifically, the PL from all these apple-based beverages were found to be less active but within the same order of magnitude when compared to the PL from wine [8] and beer [9,16] against PAF inflammatory and platelet aggregation pathways. Moreover, the activities of PL from both apple juice and cider samples against the PAF pathways are of similar potency with previously reported ones for PL extracts derived from tea [17] and from several other food sources, such as olive oil and fish [2,12,18].

These PL samples were further tested against the ADP-induced platelet aggregation, showing again potent antiplatelet effects against this pathway too, which was also comparable to previously reported ones for other plant (tea)- and marine-derived food sources (salmon, herring and boarfish) [17,18].

Inhibitory action of PL was stronger against the PAF pathway of platelet aggregation; however, for PL from samples AA, CC, AD and CA; strong inhibition can also be observed against ADP-induced platelet aggregation. Instead, the anti-platelet properties of the PL from apple juice AB and AE and from their related cider samples CB and CE against the PAF-induced platelet aggregation were significantly stronger than their effects against platelet aggregation induced by the ADP pathway.

The PL from the AA sample (low in tannins) showed the greatest inhibitory effects against both ADP- and PAF-induced platelet aggregation, and thus the stronger anti-platelet potency. Subsequently, the other apple juice and cider samples showed lower anti-platelet potency than that of apple juice AA samples, which, however, were similar within all these samples and of considerable anti-PAF and anti-ADP potency in comparison to PL from beer and other food sources [9,17,18]. Thus, apart from apple juice AA, in all the other apple juices and their related cider products, there is little to no alteration of the lipid-related bioactivities through the fermentation process in these apple products.

The most potent anti-PAF and anti-ADP activities that were observed in the PL from apple juice AA were of similar potency, since these extracts showed similar IC50 values against both these pathways of platelet aggregation. Moreover, both anti-PAF and anti-ADP potency of the PL from its fermented product, apple cider CA, were lower than that of apple juice AA, but again the anti-ADP activity for the CA PL was found to be of similar potency with its anti-PAF effects. These results showed that, in contrast to all the other apple products, fermentation of apple juice AA from Jonagold varieties affects their PL-related anti-platelet bioactivities against platelet aggregation, an effect that was of similar magnitude against both the different PAF and ADP pathways of platelet aggregation. These alterations may be related to changes in the lipid content during the fermentation process, as was previously described that this occurs in other fermented products [8,9,16,20].

Moreover, the biological activity of the PL from commercial cider sample CC and commercial apple juice sample AD showed similar effects against human platelet aggregation induced by PAF and ADP, suggesting that the pasteurization process for cider (67–68 °C for 20 min) and for apple juice (70–72 °C for 20 min) has no function in adjusting the PL-related bioactivities against different pathways of platelet aggregation in these samples.

Since PL from apple juice samples AA showed the most potent anti-platelet properties against platelet aggregation induced by either PAF or ADP, and since these effects were of similar potency, something that was also observed in the PL from its fermented product CA and from the control apple juice AD and cider CC, the PL from all these samples were further separated into molecular subclasses-fractions using HPLC analysis. Commercial apple juice AD and cider CC were used as standards in such an analysis. For all these PL extracts, HPLC analysis was performed in a wavelength of 208 nm, where double bonds in lipids, and thus the lipids themselves, can be detected, but also in 280 nm where phenolic groups usually are detected.

**Figure 4:**
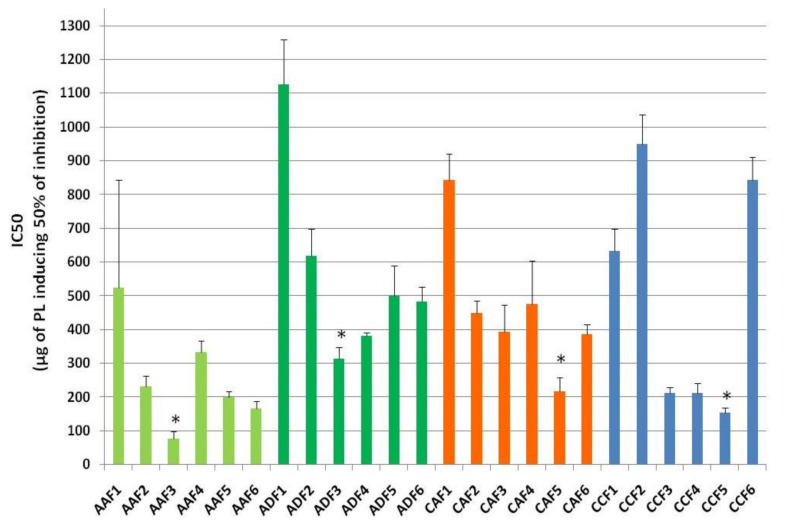
Anti-platelet potency (IC50 values) of HPLC-derived fractions of TPL from apple juice AA (light-green-coloured bars) and AD (dark-green-coloured bars), and cider samples CA (red-coloured bars) and cider CC (blue-coloured bars) against PAF-induced human platelet aggregation. Abbreviations: * denotes statistically significant difference (*p* < 0.05) within the same source; F: Fraction; AA and CA: apple juice and cider from apples with low content in tannins (Jonagold), respectively; AD: commercially found apple juice (relevant to the apple juice AA of the Jonagold variety); CC: commercially found cider ‘*Con’s Irish Cider*^®^’ (produced by fermentation of a mixture of the aforementioned three different apple juice types in a ratio of 55% Jonagold, 35% Dabinett and 10% Aston bitter).

The HPLC fractions were collected according to retention times obtained for assessed relative standards (Figure 1, Table 1). In addition, the in vitro antithrombotic activities of all fractions derived from the HPLC analysis of the PL from apple juice AA and its fermented product cider AC, but also from the commercial apple juice AD and cider CC, were further assessed for their putative anti-platelet ability to inhibit PAF-induced platelet aggregation. The results are depicted in Figure 4 and are again expressed in IC50 values (half-maximal inhibitory concentrations; μg of lipid sample needed for 50% of inhibition of platelet aggregation) for each lipid-fraction against the PAF pathway.

According to the HPLC analysis and according to the retention times of the relative standards analysed, as shown in Figure 1 and Table 1, fraction 1 compounds comprising of molecules with phenolic groups were eluted at 0–15 min. Polar lipids such as polar glyco-lipids were seen to elute in fraction 2 at 15–30 min. Fraction 3 at 30–45 min contained phosphatidilcholine (PC) molecules, while in fraction 4 the sphyngomyelin (SM) and some sulpho and glyco-lipids were eluted at 45–60 min. Fraction 5 contained phosphatidylethanolamine (PE) molecules eluted at 60–75 min, while the remaining fraction 6 was comprised of remnants of the lipid molecules previously eluted (75–90 min).

From all HPLC fractions assessed against platelet aggregation induced by PAF, some showed potent anti-PAF effects while others showed poor inhibitory action against platelet aggregation induced by PAF. More specifically, the IC50 values obtained for all HPLC fractions of the TPL extracts of these apple products when assessed against platelet aggregation, induced by PAF, showed that, in both the apple juices AA and AD, the lipid molecules eluted in the HPLC-fractions F3 of their PL extracts, where molecules of the PC family are usually eluted, were the ones with the most potent anti-PAF effects. In contrast, in both ciders CA and CD, the lipid molecules with the most potent anti-PAF activities were those eluted in the HPLC fractions F5 of their PL extracts, where molecules of the PE family are usually eluted. These results further support the notion that fermentation alters the composition and bio-functionality of the bioactive PL in apple-based beverages.

Furthermore, in all samples tested, the fractions F1 corresponding to phenolic compounds of the PL in all sources were the ones with the lowest anti-PAF activity, with the exception of cider CC, in which the fractions F2 and F6 of its PL extract exhibited also very low anti-PAF potency.

Overall, of all the fractions, the weakest bio-activity was seen in fraction 1, followed by fractions 2, 6, 4, and finally fraction 3 of PC was the most bio-active in PL from apple juices, and fraction 5 of PE was the most bio-active in PL from cider samples, respectively.

Moreover, from all fractions tested, the most potent anti-PAF effects were observed in fraction 3 from PL of apple juice AA, with IC50 values of 50–100 μg, which are in accordance with similar outcomes in bioactive PC and PE fractions in wine, beer and other fermented products [8,9,12,20]. In addition, these results might explain the strongest anti-PAF effects observed in the PL of this apple juice sample in comparison to the ones of the PL from all the other apple juice and cider samples.

Nonetheless, it must be noted that, when working in collusion, the inhibitory effect of all fractions of the PL when interacting with the PAF/PAF-R (platelet activating factor/platelet activating factor receptor) pathway was seen to be much stronger, which cannot be attained individually by each lipid fraction, rather from a synergism of all these PL molecular species co-existing within a PL extract. Such pattern of synergistic effects of different fractions—molecular species of PL—when co-presented in the overall TPL extracts have been previously reported for other food sources and fermented products too [8,9,12,20,21].

### 3.3. Fatty Acid Composition of Bioactive PL from Apple Juices and Their Fermented Cider Products

The fatty acid profiles of the bioactive PL extracts for each of the apple juices and cider samples obtained from their GCMS analysis are shown in Table 3., while for the most bioactive PC and PE subclasses (HPLC-fractions) in Table 4.

PL of all these apple products were found to be rich in polyunsaturated fatty acids (PUFA). More specifically, in apple juices AA, AD and AE, as well as the cider products CA and CB, PUFA were the most abundant fatty acid class present, accounting approximately for the 55–75% of the total fatty acids, which were followed by saturated fatty acids (SFA) and monounsaturated fatty acids (MUFA) content (Table 3). The TPL from apple juice AB and ciders CE and CC were again rich in PUFA, which however accounted less in the total fatty acids, approximately 30–50%, which was also found to be approximately similar with the relative levels of the SFA, followed by less but considerable amounts of the MUFA content in these samples (Table 4).

The most abundant PUFA from all samples of the PL extracts was omega-6 (n6) linoleic acid (LA; 18:2 n6) as well as omega-3 (n3) alpha linolenic acid (ALA; 18:3 n3). The most abundant MUFA was seen to be omega-9 (n9) oleic acid (OA; 18:1 c9), followed by n9 eicosenoic acid (20:1 c11). In the SFA, palmitic acid (16:0), followed by stearic acid (18:0), were the most abundant.

These results concerning the fatty acid composition of PL from apple juice and apple cider are in accordance with previously reported ones for lipids from apple juice and cider [22,23], but also in PL from other plant-derived food sources used for beverages products, such as beer and tea [9,17].

The relative omega-6 polyunsaturated fatty acids (n6PUFA) content of the PL from the unfermented raw apple juices AA were higher and the omega-3 polyunsaturated fatty acids (n3PUFA) content was lower when compared with the relative n6 and n3 PUFA contents of the PL from the cider A sample. Thus, the n6/n3 ratio of TPL from the fermented cider sample CA was somewhat lower than the ratio for apple juice sample AA.

On the other hand, even though the relative n6PUFA content of the PL from the unfermented raw apple juices AB was similar to that of its fermented product CB, the observed increase in the PUFA content in CB can be attributed to a significant increase on its n3PUFA content, and thus the omega-6/omega-3 PUFA (n6/n3 PUFA) ratio of the CB cider was also found to be lower than the relative n6/n3 ratio for its unfermented raw apple juice AB.

Furthermore, for both apple juice AE and its fermented-type cider CE, the PUFA content of the PL form cider CE was lower than that of the PL from apple juice AE, while the SFA and MUFA contents from the PL of cider CE were higher than that of apple juice AE. Subsequently, the relative ratio of the omega-6/omega-3 PUFA (n6/n3 PUFA) for both AE and CE samples was found to be similar.

For the commercial apple juice, although PUFA was the most abundant FA, it had the highest n6 PUFA content and therefore the highest n6/n3 ratio. Conversely, the TPL of the commercial cider sample contained the lowest amount of PUFA and subsequently had the lowest n6/n3 ratio.

Independently of the alterations observed in the n6/n3 PUFA content and ratios, the bioactive PL from all these apple products were found to contain a favorable ratio of n6/n3 lower than 5, with PL from apple juices exhibiting a n6/n3 ratio within the range of 3–5 and PL from cider samples exhibiting a n6/n3 ratio within the range of 0.5–2.5, approximately. These results show that there is a favorable reduction of this ratio due to fermentation, but most importantly, all these values for the n6/n3 ratio in all samples assessed were found within a range for this ratio that is usually observed in healthy foods and diets, and much lower than values above 15/1 for this ratio that are usually observed in unfavorable western style foods and diets [24].

In addition, for the bioactive PL fractions of PC for apple juices AA and AD and PE for apple ciders CA and CC, the SFA were the most abundant class of fatty acids followed by PUFA and MUFA. The most abundant SFA again were palmitic acid (16:0) and stearic acid (18:0)t. Also, the most abundant PUFA from all these bioactive PL fractions were n6 linoleic acid (LA; 18:2 n6) and the n3 alpha linolenic acid (ALA; 18:3 n3), while the most abundant MUFA was again the n9 oleic acid (OA; 18:1 c9).

The relative n3PUFA contents of all these bioactive PL-fractions were much higher than their n3PUFA content. Subsequently, the n6/n3 ratio of all these bioactive PL-fractions, PC in apple juices and PE in apple cider products, had values ranging between 0.16 and 0.57, which were lower than the value of 1 and even much lower than the relative ratios of the PL of all apple products. These results come in accordance with previous outcomes in other plant-derived beverages and fermented products like beer [9], while they show more profoundly that, in the apple products assessed, bioactive PL subclasses exist, such as the bioactive PC and PE, with very low values for their n6/n3 ratio that are usually observed in healthy foods and diets, and much lower than values above 15/1 for this ratio that are usually observed in unfavourable western style foods and diets [24].

It has been proposed that, the lower the levels for this n6/n3 PUFA ration in foods and diets, the better the preventative outcome against inflammation and platelet aggregation-related chronic disorders [24]. The above findings of the low n6/n3 PUFA ratios in all the apple products tested, and especially in their bioactive PL subclasses of PC and PE, further support the anti-inflammatory and cardio-protective properties of the apple juices and cider PL.

Furthermore, the presence of polar lipid compounds in apple products that are rich in n3 PUFA, like the bioactive PC and PE fractions in apple juices and ciders, also enhance their cardio-protective potency, since such bioactive compounds were found to be the ones with the highest anti-inflammatory and antithrombotic potency against the PAF pathway of platelet aggregation and of leukocyte and endothelial/mesangial cells activation in several other foods, beverages and fermented products [9,12,20,21,25,26], while only recently a standard PC containing n3 PUFA was found to possess strong anti-platelet and antithrombotic properties against the PAF, ADP, thrombin and collagen pathways, with higher specificity against the PAF pathway, which were comparable to that of specific antiplatelet drugs such as aspirin and ginkgolides [17].

## 4. Conclusions

This is the first study exhibiting that Irish apple juice and its fermented product, Real Irish cider, contain bioactive PL compounds with strong antiplatelet activities against the inflammatory and thrombotic mediator PAF and the well-established platelet agonist ADP. These anti-platelet properties were found to be comparable with those observed in polar lipids from several other healthy food sources. HPLC and GC–MS analysis facilitated the detection of changes in the bioactivities, lipid content and fatty acid composition observed due to fermentation. HPLC fractions of PC molecules exhibited the strongest anti-platelet effects in apple juices, while PE molecules were more bioactive in cider products. Even though the HPLC fractions individually showed lowest antithrombotic properties compared to their PL extracts, when these bioactive PL fractions are present and working collectively in their PL-extract, they thus showed synergistically stronger anti-PAF activities and inhibition of platelet aggregation.

In addition, the PL from all these apple product sources were found to be rich in PUFA, and especially beneficial in n-3 PUFA, but also in MUFA, providing a rationale for their strong anti-inflammatory and anti-platelet properties, while they also exhibited favorable cardio-protective levels of the n6/n3 PUFA ratio, especially for the bioactive PC and PE subclasses, with fermentation providing lower values for this ratio in the PL of the fermented cider products.

Further studies are needed in order to fully investigate the antiplatelet and anti-inflammatory benefits of fresh Irish apple juice and its fermented product, Real Irish Cider, as well as the benefits and effects of the fermentation process; nonetheless, so far, results from this research are promising.

## Figures and Tables

**Table 1:** Retention Times (RTs) of Individual Standards assessed during HPLC.

Peak	Standards	Molecular Class	RT (min)
1	Epicatechin (3 mg/mL)	Phenolic	2–4 (280 nm)
2	Theophylline (3 mg/mL)	Phenolic	3–5 (280 nm)
3	Gallic Acid (3 mg/mL)	Phenolic	5–8 (280 nm)
4	Quercetin (3 mg/mL)	Phenolic	10–16 (280 nm)
5	Cerebrosides (0.5 mg/mL)	Glycosphingolipid	25–30 (208 nm)
6	L-α-Phosphatidylcholine (1.5 mg/mL)	Glycerol-based phospholipid	35–40 (208 nm)
7	Sphyngomyelin (0.3 mg/mL)	sphingosine-based phospholipid	45–50 (208 nm)
8	L-α-Lysophosphatidylcholine from Glycine max (soybean) (0.3 mg/mL)	Glycerol-based phospholipid	50–53 (208 nm)
9	Digalactosyldiglyceride (0.3 mg/mL)	Glycerol-based Glycolipid	53–56 (208 nm)
10	Sulfatides (0.3 mg/mL)	Glycerol-based Glyco-Sulfo-lipid	56–59 (208 nm)
11	L-α-Phosphatidylethanolamine from Glycine max (soybean) (1.2 mg/mL)	Glycerol-based phospholipid	62–67 (208 nm)
12	L-α-Phosphatidylinositol sodium salt from Glycine max (soybean) (0.9 mg/mL)	Glycerol-based phospholipid	75–78 (208 nm)

**Table 2:** The yield of extraction for the total lipids (TL), neutral lipids (NL) and polar lipids (PL) expressed as g/100 g of sample (mean ± SD, n = 3).

Samples	TL (g/100 g)	NL (g/100 g)	PL (g/100 g)
Apple Juice AA	0.072 ± 0.002	0.016 ± 0.002	0.056 ± 0.004
Apple Juice AB	0.042 ± 0.005	0.010 ± 0.005	0.031 ± 0.003
Apple Juice AE	0.221 ± 0.143	0.030 ± 0.035	0.191 ± 0.116
Commercial Apple Juice AD	0.150 ± 0.051	0.070 ± 0.058	0.080 ± 0.016
Cider CA	0.033 ± 0.012	0.005 ± 0.002	0.028 ± 0.004
Cider CB	0.025 ± 0.009	0.004 ± 0.001	0.021 ± 0.003
Cider CE	0.046 ± 0.013	0.006 ± 0.001	0.040 ± 0.005
Commercial Cider CC	0.042 ± 0.005	0.007 ± 0.003	0.035 ± 0.002

Abbreviations: TL: Total lipids; NL: Neutral lipids; AA and CA: apple juice and cider from apples with low content in tannins (Jonagold), respectively; AB and CB: apple juice and cider from apples with moderate content in tannins (Dabinett); AE and CE: apple juice and cider from apples with high content in tannins (Aston Bitter); AD: commercially found apple juice (relevant to the apple juice AA of the Jonagold variety); CC: commercially found cider ‘Con’s Irish Cider^®^’ (produced by fermentation of a mixture of the aforementioned three different apple juice types in a ratio of 55% Jonagold, 35% Dabinett and 10% Aston bitter).

**Table 3:** The fatty acid profile obtained of the polar lipid extracts of the apple juice and apple cider samples expressed as the percentage of each fatty acid in the total fatty acids for each sample (mean ± SD, n = 3).

Fatty Acid	AA	AB	AD	AE	CA	CB	CE	CC
C12:0	0.10 ± 0.04	ND	ND	ND	0.417 ± 0.246	ND	0.64 ± 0.06	ND
C14:0	2.46 ± 0.32	3.824 ± 0.845	0.648 ± 0.183	0.147 ± 0.054	1.637 ± 1.056	ND	1.56 ± 0.09	4.52 ± 0.48
C15:0	0.28 ± 0.02	0.383 ± 0.118	0.12 ± 0.033	0.118 ± 0.014	0.179 ± 0.119	ND	0.22 ± 0.02	0.45 ± 0.12
C16:0	21.83 ± 2.43	24.928 ± 2.95	14.39 ± 1.62	17.38 ± 0.88	15.80 ± 6.88	10.05 ± 3.71	20.62 ± 2.96	22.07 ± 3.38
C16:1 c9	0.55 ± 0.12	1.32 ± 0.22	0.09 ± 0.01	0.19 ± 0.05	0.30 ± 0.17	0.13 ± 0.30	1.03 ± 0.12	0.39 ± 0.33
C17:0	0.43 ± 0.10	0.51 ± 0.03	0.38 ± 0.05	0.39 ± 0.07	0.22 ± 0.27	0.87 ± 1.51	0.39 ± 0.08	0.58 ± 0.81
C18:0	5.57 ± 0.08	9.42 ± 0.26	3.58 ± 0.03	4.63 ± 0.44	6.64 ± 1.51	4.34 ± 0.43	5.80 ± 0.64	9.04 ± 1.39
C18:1 c9	5.71 ± 0.78	8.923 ± 0.30	3.82 ± 0.07	2.01 ± 0.67	8.04 ± 1.58	2.44 ± 0.89	8.73 ± 0.93	4.14 ± 1.00
C18:2 c9,12	42.28 ± 3.71	25.81 ± 0.74	59.43 ± 1.67	36.10 ± 1.51	18.44 ± 4.84	16.21 ± 4.90	20.17 ± 3.61	10.30 ± 1.41
C18:3 c9,12,15	8.07 ± 1.31	6.39 ± 0.84	9.80 ± 0.32	16.80 ± 2.04	5.34 ± 2.42	8.79 ± 3.30	4.26 ± 0.55	1.61 ± 0.68
C20:0	2.60 ± 0.15	2.10 ± 0.29	3.08 ± 0.33	2.59 ± 0.34	2.956 ± 0.24	3.30 ± 1.14	3.26 ± 0.08	2.56 ± 1.25
C20:1 c11	1.42 ± 0.17	3.94 ± 0.23	0.74 ± 0.12	1.54 ± 0.26	2.25 ± 1.21	6.21 ± 2.16	3.17 ± 0.57	2.63 ± 1.27
C20:2 c11,14	0.51 ± 0.14	0.53 ± 0.18	0.51 ± 0.40	0.72 ± 0.07	0.87 ± 0.35	2.44 ± 1.04	0.47 ± 0.16	1.34 ± 0.52
C20:3 c8,11,14	0.23 ± 0.15	0.15 ± 0.03	0.09 ± 0.03	0.30 ± 0.14	0.67 ± 0.15	1.10 ± 0.09	0.36 ± 0.22	2.20 ± 0.82
C20:4 c5,8,11,14	0.92 ± 0.38	0.21 ± 0.12	0.15 ± 0.12	0.05 ± 0.01	2.87 ± 2.42	2.57 ± 2.60	0.08 ± 0.05	2.19 ± 2.24
C20:5 c5,11,14,17	0.40 ± 0.34	0.22 ± 0.12	0.07 ± 0.02	0.11 ± 0.09	0.49 ± 0.39	4.66 ± 4.23	0.11 ± 0.08	3.51 ± 3.98
C22:0	0.29 ± 0.12	0.25 ± 0.05	0.08 ± 0.09	0.23 ± 0.12	1.37 ± 1.20	4.88 ± 3.83	1.33 ± 0.53	2.90 ± 1.06
C20:4 c5,8,11,14	0.46 ± 0.39	0.19 ± 0.16	0.17 ± 0.10	2.56 ± 0.92	2.99 ± 1.09	2.52 ± 1.79	0.32 ± 0.22	3.23 ± 1.51
C20:5 c5,8,11,14,17	1.64 ± 0.41	1.83 ± 1.03	2.02 ± 0.73	0.50 ± 0.37	5.26 ± 1.71	5.08 ± 4.53	2.69 ± 0.58	4.38 ± 1.31
C22:1 c13	1.94 ± 0.31	4.96 ± 0.09	0.10 ± 0.06	3.89 ± 0.71	4.92 ± 3.11	6.06 ± 3.10	20.2 ± 9.08	4.12 ± 1.01
C22:4 c7,10,13,16	0.46 ± 0.23	0.77 ± 0.24	0.22 ± 0.03	4.22 ± 4.18	8.47 ± 4.89	8.10 ± 2.84	1.76 ± 0.93	6.40 ± 2.14
C22:5 c7,10,13,16,19	1.25 ± 0.58	1.86 ± 0.88	0.31 ± 0.064	3.32 ± 0.71	2.49 ± 1.27	5.10 ± 1.82	1.67 ± 0.81	7.24 ± 3.14
C22:6 c4,7,10,13,16,19	0.62 ± 0.71	1.49 ± 1.95	0.25 ± 0.12	1.43 ± 0.73	7.41 ± 3.61	5.17 ± 1.45	1.16 ± 0.39	4.69 ± 1.81
SFA	33.54 ± 2.52	41.41 ± 2.99	22.27 ± 1.64	25.48 ± 1.68	29.22 ± 8.79	23.44 ± 0.83	33.85 ± 3.70	41.76 ± 3.34
MUFA	9.62 ± 0.90	19.14 ± 0.48	4.75 ± 0.10	7.64 ± 1.14	15.50 ± 2.83	14.84 ± 3.86	33.11 ± 7.53	11.14 ± 3.15
PUFA	56.84 ± 2.48	39.45 ± 3.38	72.98 ± 1.73	66.99 ± 2.72	55.29 ± 5.97	61.73 ± 4.43	33.04 ± 3.95	47.10 ± 5.54
n6	44.40 ± 3.34	27.47 ± 0.74	60.37 ± 1.52	42.28 ± 2.93	31.32 ± 3.12	30.40 ± 5.06	22.84 ± 3.54	22.43 ± 2.86
n3	12.44 ± 1.22	11.98 ± 3.96	12.61 ± 0.31	24.71 ± 0.28	23.97 ± 3.14	31.33 ± 8.92	10.21 ± 0.50	24.66 ± 6.07
n6/n3	3.61 ± 0.64	2.46 ± 0.88	4.79 ± 0.24	1.71 ± 0.14	1.31 ± 0.30	1.08 ± 0.49	2.23 ± 0.46	0.96 ± 0.36

Abbreviations: AA and CA: apple juice and cider from apples with low content in tannins (Jonagold), respectively; AB and CB: apple juice and cider from apples with moderate content in tannins (*Dabinett*); AE and CE: apple juice and cider from apples with high content in tannins (*Aston Bitter*); AD: commercially found apple juice (relevant to the apple juice AA of the Jonagold variety); CC: commercially found cider ‘*Con’s Irish Cider*^®^’ (produced by fermentation of a mixture of the aforementioned three different apple juice types in a ratio of 55% Jonagold, 35% Dabinett and 10% Aston bitter); c: cis; SFA: saturated fatty acids; MUFA: monounsaturated fatty acids; PUFA: polyunsaturated fatty acids; n3: omega-3 PUFA; n6: omega-6 PUFA; ND: non-detectable.

**Table 4:** The fatty acid profile obtained of the bioactive polar lipid fractions of PC and PE of the apple juice and apple cider samples expressed as the percentage of each fatty acid in the total fatty acids for each sample (mean ± SD, n = 3).

Fatty Acid	Fraction 3 (PC) of AA	Fraction 3 (PC) of AD	Fraction 5 (PE) of CA	Fraction 5 (PE) of CC
C12:0	1.45 ± 0.12	ND	ND	ND
C14:0	3.36 ± 0.10	4.40 ± 0.79	7.24 ± 2.61	4.34 ± 0.97
C15:0	3.36 ± 0.12	ND	ND	ND
C16:0	24.55 ± 4.28	28.08 ± 3.61	30.75 ± 6.52	31.41 ± 5.88
C16:1 c9	2.65 ± 0.30	2.27 ± 0.20	3.45 ± 0.10	1.06 ± 0.15
C17:0	1.27 ± 0.12	ND	ND	ND
C18:0	33.33 ± 8.61	19.20 ± 1.05	24.39 ± 3.79	18.72 ± 2.67
C18:1 c9	5.31 ± 1.90	13.33 ± 2.81	15.36 ± 2.91	14.17 ± 3.60
C18:2 c9,12	2.54 ± 2.04	5.80 ± 1.42	4.43 ± 1.02	5.80 ± 4.32
C18:3 c9,12,15	5.17 ± 0.85	5.13 ± 3.78	4.06 ± 1.02	3.85 ± 2.19
C20:0	2.93 ± 2.89	2.17 ± 0.86	1.27 ± 0.70	2.51 ± 1.25
C20:1 c11	3.78 ± 0.90	3.60 ± 1.08	0.85 ± 0.37	2.28 ± 1.24
C20:5 c5,8,11,14,17	2.17 ± 0.71	3.25 ± 0.60	1.36 ± 0.63	2.53 ± 1.19
C22:1 c13	3.69 ± 1.19	2.81 ± 0.91	1.55 ± 0.90	3.98 ± 1.14
C22:5 c7,10,13,16,19	3.74 ± 1.56	4.98 ± 1.58	2.29 ± 0.95	3.60 ± 0.97
C22:6 c4,7,10,13,16,19	3.84 ± 1.05	4.98 ± 0.30	1.57 ± 0.27	2.57 ± 1.54
SFA	67.10 ± 6.69	53.85 ± 6.32	60.81 ± 12.60	56.98 ± 4.64
MUFA	15.43 ± 2.89	22.01 ± 2.31	19.77 ± 3.36	21.49 ± 4.87
PUFA	17.47 ± 5.44	24.14 ± 5.44	13.71 ± 3.40	22.33 ± 1.26
n6	2.54 ± 2.03	5.80 ± 1.42	4.43 ± 1.02	5.80 ± 4.45
n3	14.93 ± 3.49	18.34 ± 4.06	9.28 ± 2.57	12.55 ± 3.67
n6/n3	0.16 ± 0.16	0.32 ± 0.14	0.49 ± 0.24	0.57 ± 0.61

Abbreviations: AA and CA: apple juice and cider from apples with low content in tannins (Jonagold), respectively; AD: commercially found apple juice (relevant to the apple juice AA of the Jonagold variety); CC: commercially found cider ‘*Con’s Irish Cider*^®^’ (produced by fermentation of a mixture of the aforementioned three different apple juice types in a ratio of 55% Jonagold, 35% Dabinett and 10% Aston bitter); PC: Phosphatidylcholine; PE: Phosphatidylethanolamine; c: cis; SFA: saturated fatty acids; MUFA: monounsaturated fatty acids; PUFA: polyunsaturated fatty acids; n3: omega-3 PUFA; n6: omega-6 PUFA; ND: non-detectable.

## Abbreviations

AA and CA	apple juice and cider from apples with low content in tannins (Jonagold), respectively
AB and CB	apple juice and cider from apples with moderate content in tannins (Dabinett)
AE and CE	apple juice and cider from apples with high content in tannins (Aston Bitter)
AD	commercially found apple juice (relevant to and produced by pasteurization of the apple juice AA of the Jonagold variety)
CC	commercially found cider ‘Con’s Irish Cider^®^’ (produced by fermentation of a mixture of the aforementioned three different apple juice types in a ratio of 55% Jonagold, 35% Dabinett and 10% Aston bitter)
PL	polar lipids
TL	total lipids
NL	neutral lipids
HPLC	high-performance liquid chromatography
GCMS	gas chromatography mass spectra
PC	phosphatidylcholine
SM	sphyngomyelin
PE	phosphatidylethanolamine
PI	phosphatidylinositol
SFA	saturated fatty acids
MUFA	monounsaturated fatty acids
PUFA	polyunsaturated fatty acids
n3	omega-3 PUFA
n6	omega-6 PUFA
ND	non-detectable
